# Effect of Aqueous Extract from *Morinda officinalis F. C. How* on Microwave-Induced Hypothalamic-Pituitary-Testis Axis Impairment in Male Sprague-Dawley Rats

**DOI:** 10.1155/2015/360730

**Published:** 2015-09-08

**Authors:** Bin Song, Fengjuan Wang, Wei Wang

**Affiliations:** ^1^Department of Human Anatomy and Histoembryology, School of Basic Medical Sciences, Fujian Medical University, Fuzhou 350108, China; ^2^Key Laboratory of Brain Aging and Neurodegenerative Diseases of Fujian Provincial Universities and Colleges, Fuzhou 350108, China; ^3^Research Center for Neuroscience, Fujian Medical University, Fuzhou 350108, China; ^4^Department of Breast Surgery, First Ailiated Hospital of Xiamen University, Xiamen 361003, China

## Abstract

The present study aimed to assess the protective effects of aqueous extract from *Morinda officinalis F. C. How *on microwave-induced reproductive impairment in male rats. Microwave exposure injury was induced by exposure of 900 MHz microwaves at 218 *μ*m/cm^2^radiation densities, 24 hours/day for 10 days. Male Sprague-Dawley rats were randomized to: normal control, microwave exposure model, or water layer or ethyl acetate layer of aqueous extract 40 g/kg treatment groups. After 2 weeks of treatment, sexual performance, serum levels of gonadotrophin-releasing hormone (GnRH), luteinizing hormone (LH), follicle-stimulating hormone (FSH) or testosterone, morphological analysis of testis and epididymis, and GnRH protein expression in the hypothalamus were measured. Pretreatment with water layer of aqueous extract 40 g/kg significantly improved sexual performance, increased serum testosterone level, and decreased LH and GnRH level compared with microwave exposed model rats (all *P* < 0.05). Water layer of aqueous extract treatment significantly increased seminiferous cell or sperm number in testis and epididymis. Protein expression of GnRH in the hypothalamus significantly decreased in the water layer of aqueous extract treated group (*P* < 0.05). Ethyl acetate layer of aqueous extract did not show obvious effects on the measured parameters. These findings suggest that water layer of aqueous extract 40 g/kg ameliorates microwave-reduced reproductive impairment.

## 1. Introduction

Male infertility is a global population health concern. Approximately 15% of couples have difficulty conceiving, and male factor contributes to 40–60% of those cases [1, 2]. The most common cause of male infertility is loss of sufficient normal, active sperm [3]. Microwaves from mobile phones can be added to the environmental factors that contribute to male infertility [4, 5]. Exposure to cell phone microwaves may decrease sperm parameters [6]. Therefore, prevention and treatment of microwave-induced male reproductive impairments urgently need to be addressed.

The underlying mechanisms of microwave-induced reproductive impairment in males are largely unknown. The hypothalamic-pituitary-testicular axis represents a complex neuroendocrine feedback loop regulating reproductive function. Gonadotropin-releasing hormone (GnRH), a hormone produced by specific neurons in the hypothalamus, forms the final common pathway regulating reproductive function [7]. GnRH induces synthesis and secretion of the luteinizing hormone (LH) and follicle-stimulating hormone (FSH). FSH is responsible for the activation of the seminiferous tubules and production of sperm. Testicular function is controlled by the central nervous system* via* FSH and LH. Testosterone is positively linked with sexual intimacy in men [8] and plays a significant role in spermatogenesis in adult males.

The male reproductive system is highly susceptible to microwave radiations [9]. Microwaves from mobile phones located nearby the testicular organs can penetrate testicular tissues. In addition, the central nervous system is susceptible to microwave exposure [10, 11]. Microwaves may have both thermal and nonthermal effects on the reproductive tissues [12], which may lead to decreased sperm count, enzymatic and hormonal changes, DNA damage, and apoptosis formation [13].

There have been many attempts to reduce adverse effects of microwave radiations through use of herbal drugs. The root of* Morinda officinalis F. C. How* is widely used for the treatment of sexual dysfunctions in China and has a potential role in the maintenance of male reproduction. Treatment with crude drug of* Morinda officinalis F. C. How* increased sperm activity and sperm density [14]. Both aqueous and alcohol extracts of* Morinda officinalis F. C. How* could promote spermatogenesis and repair reproductive injury induced by microwave radiation [15, 16]. Many bioactive constituents are isolated from* Morinda officinalis F. C. How* such as anthraquinone, carbohydrate constituents, beta-sitosterol, and ethylcholesterol [17]. The invigorating Kidney-Yang effect of* Morinda officinalis F. C. How* derives mainly from the fraction of water and ethyl acetate solvent extraction [18]. However, the effective fraction of* Morinda officinalis F. C. How* against microwave-induced reproductive impairment is not clear.

We aimed to investigate the protective effects of ethyl acetate layer and water layer fraction of aqueous extract derived from* Morinda officinalis F. C. How* on microwave-induced reproductive impairment in male rats.

## 2. Material and Methods

### 2.1. Materials

Twenty-four male healthy Sprague-Dawley rats (4 months old, weighing 247.7 ± 13.6 g) were obtained from the Animal Experiment Center of Fujian Medical University (certificate number: SCXK [Min] 2012-0001). The rats were housed in a temperature controlled room (18°C–24°C) and 45–65% humidity on a 12 h light and 12 h darkness cycle. All rats were fed a standard diet and had free access to water. The dried roots of* Morinda officinalis F. C. How* were purchased from the rural commercial association of Yongding, China, and the plant was authenticated by herbal expert L. Zheng of rural commercial association of Yongding. The GnRH, LH, FSH, and testosterone enzyme linked immunosorbent assay (ELISA) kits were all purchased from R&D Co. Ltd. Rabbit polyclonal anti-GnRH (ab5617) was purchased from Abcam Biological Products Co. Ltd. Donkey anti-rabbit green fluorescent antibody (A21206) was from Invitrogen Co. Ltd. of USA and 4,6-diamidino-2-phenylindole (DAPI) was obtained from Sigma-Aldrich Co. Ltd.

### 2.2. Preparation of Aqueous Extract Derived from* Morinda officinalis F. C. How*

**Figure 1:**
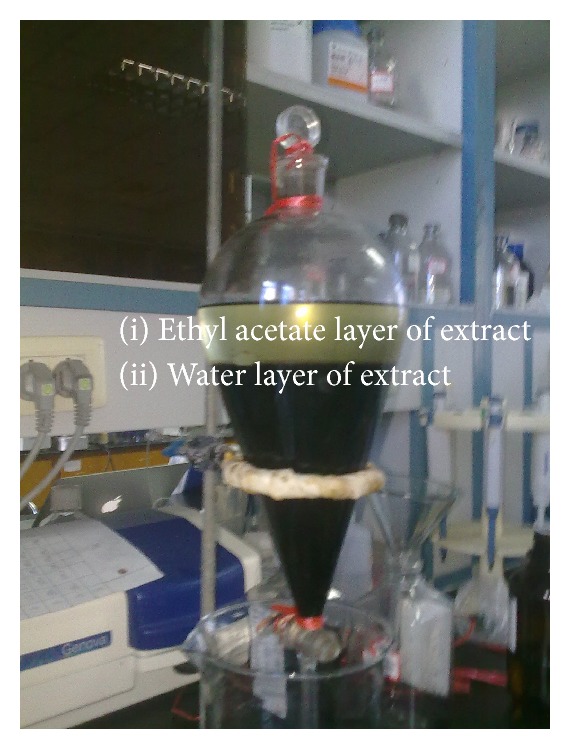
Water layer and ethyl acetate layer fraction of aqueous extract derived from* Morinda officinalis F. C. How*. The upper shallow yellow solution is the ethyl acetate layer fraction; lower dark brown solution is the water layer fraction.

Dried* Morinda officinalis F. C. How* roots were ground to fine powder and boiled with 10- and 6-fold volumes of water at 100°C for 2 h. The combined crude extracts suspension was then filtered with filter paper and evaporated on a rotatory evaporator under reduced pressure. The condensed extract, 500 mL, was mixed with 500 mL ethyl acetate in a 1,000 mL separating funnel. After 30 min of being stationary, the solution divided into 2 layers: ethyl acetate layer fraction (upper shallow yellow solution) and water layer fraction (lower dark brown solution) of the aqueous extract (Figure 1). Finally, the water layer or ethyl acetate layer fraction was collected separately for the subsequent experiments. In this paper, the dose of ethyl acetate layer or water layer fraction of aqueous extract 40 g/kg was expressed in terms of dried weight of the plant to body weight.

### 2.3. Experimental Protocol

All experimental protocols were approved by the laboratory animal center of the Academy of Fujian Medical University and carried out by the Guidelines of Care and Use of Laboratory Animals. Twenty-four rats were randomized equally to four groups: normal control group, microwave exposure model group, microwave exposure plus ethyl acetate layer fraction 40 g/kg treatment group, and microwave exposure plus water layer fraction 40 g/kg treatment group. Rats in the treatment group were orally administered 40 g/kg ethyl acetate layer fraction or water layer fraction at the end of microwave exposure consecutively for 10 days, whereas rats in the normal control and microwave exposure model groups orally received the same volume of saline.

Rats receiving microwave exposure were placed in a Plexiglas cage (six rats at a cage). The cage was covered with a homemade tin box at 50 cm × 50 cm × 50 cm. The opening of the cage was faced by the horn antenna in a 10 cm distance. Rats were exposed to 900 MHz of continuous microwave through a microwave signal generator (Quanzhou Rongshengda telecommunications Co., China), 24 h/day for 10 days. The radiation density of the cage was 218 *μ*m/cm^2^ under a ML-91 microwave leakage tester (Suqian Jingchen radio equipment Co. Ltd., China).

### 2.4. Sample Collection and Preparation

At the end of the experimental period, rats were weighed and sacrificed under anesthesia with an intraperitoneal injection of 4–6 mg/kg 10% chloral hydrate. Blood samples (3 mL) were collected from the right atrium and then centrifuged at 2,500 r/min for 15 min. The serum was stored at −20°C for the enzyme assays. Testis and epididymis were excised and cleared of adhering connective tissues. Testis and epididymis were fixed in 4% polyformaldehyde and picric acid for histopathological analysis. Brain including hypothalamic tissues was dissected out and stored in 30% sucrose solution at 4°C for subsequent frozen section and immunofluorescence staining.

### 2.5. Sexual Performance

At the end of treatment, the experimental rats were placed in a 45 × 25 × 20 cm cage and were allowed to adapt to the cage environment for 5 min. The same number of healthy female rats was then added to the cage. Two observers separately recorded the capture incubation period (CIP) and catching frequency (CF). CIP was calculated as the time between the introductions of the female to the occurrence of first male catching a female rat. CF was observed as the total number of males catching a female rat, heterosexual mounting, or cunnilingus action within 30 min.

### 2.6. Determination of Relative Reproductive Organ Weight

The absolute weights of the testes and epididymides were weighed before being fixed in the polyformaldehyde. Sample weights relative to body weights were calculated as g/g body weight.

### 2.7. Determination of GnRH, LH, FSH, or Testosterone in the Serum

Serum GnRH, LH, FSH, or testosterone levels were measured by ELISA according to the manufacturers' instructions and expressed as ng/L. The optical density was read using a spectrophotometer (Bio-Tek ELx 800, USA) at wavelength 450 nm.

### 2.8. Histopathological Examination of Testis and Epididymis

After fixation, testis and epididymis were paraffin embedded and cut at 20 *μ*m thickness for subsequent haematoxylin and eosin (HE) staining. Histopathological changes in the testis and epididymis were analyzed using a light microscope (Olympus XSP-C203, Japan) at 200x magnitude by a histologist blinded to the sample treatments.

### 2.9. Detection of GnRH Protein Expression by Immunohistochemistry

Dual labeling immunofluorescence staining was used to determine the expression of GnRH. Briefly, the hypothalamic tissues were soaked in 30% sucrose and embedded in OCT media and then cut coronally into 20 *μ*m frozen sections under −20°C. After being washed with 0.1 M phosphate-buffered saline (PBS), tissue sections were blocked with 5% bovine serum albumin and 0.1% Triton X-100 for 30 min and then incubated overnight at 4°C with 20 *μ*L rabbit polyclonal anti-GnRH (1 : 500 dilutions). Sections were washed and incubated with 20 *μ*L donkey anti-rabbit fluorescence (1 : 200 dilutions) for 45 min at room temperature. Sections were further washed and incubated with 5 *μ*g/mL DAPI 20 *μ*L solutions for 5 min. The double stained sections were viewed under LEICA TCS SP5 confocal microscope (Leica Biosystems, Germany). For control purposes, the anti-GnRH was omitted. The presence of positive expression of GnRH was determined using the Leica LAS AF Lite software (Leica Biosystems, Germany). Six fields of view per section were randomly observed. The percentage of positive expression of GnRH was determined by counting the number of positive expressions of GnRH and divided by total number of GnRH.

### 2.10. Data Analysis

Normally distributed continuous data were expressed as mean ± standard deviation (SD). Differences between groups were determined using one-way analysis of variance (ANOVA) followed by LSD's *t*-test using SPSS software version 20.0. A *P* value less than 0.05 was considered statistically significant.

## 3. Results

### 3.1. Effects of Different Parts of Aqueous Extract on CIP and CF

**Figure 2:**
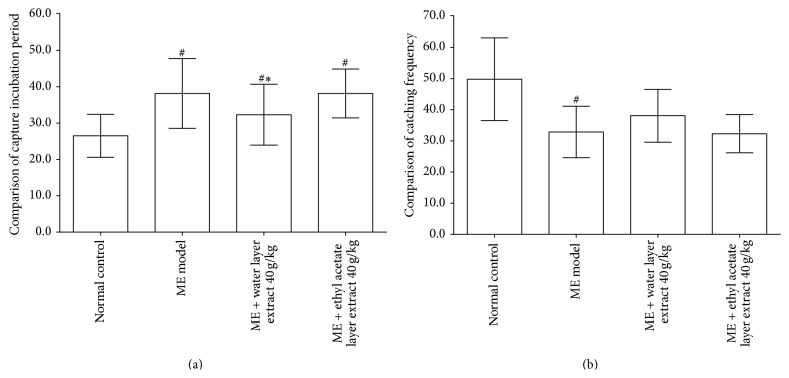
Effects of different parts of aqueous extract on capture incubation period (a) and catching frequency (b). Compared with the microwave exposure model group, water layer fraction of aqueous extract 40 g/kg treated rats showed a decreased capture incubation period and an increased catching frequency (all *P* < 0.05). Above changes were not observed in the ethyl acetate layer fraction of aqueous extract 40 g/kg treated rats. Values are expressed as mean ± SD (*n* = 6). ^#^ *P* < 0.05,* versus* normal control group. ^*∗*^ *P* < 0.05,* versus* microwave exposure model group. ME = microwave exposure.

As shown in Figure 2(a), significant increases in the CIP were observed in the microwave exposure model compared with normal control (38.1667 ± 4.8751* versus* 26.500 ± 3.0166 sec; *P* < 0.05). Treatment with water layer fraction of aqueous extract 40 g/kg decreased CIP compared with the microwave exposure model group (32.500 ± 4.1833* versus* 38.1667 ± 4.8751 sec; *P* < 0.05). There was no statistically significant difference in CIP (38.1667 ± 3.37145* versus* 39.1667 ± 3.1885 sec, *P* > 0.05) between ethyl acetate layer fraction of aqueous extract 40 g/kg and microwave exposure group.

As shown in Figure 2(b), CF of the microwave exposure group was significantly lower compared with the normal control group (32.6667 ± 4.1793* versus* 49.6667 ± 6.4705 times, *P* < 0.05). Treatment with water layer fraction of aqueous extract 40 g/kg showed a trend to increase CF compared with the microwave exposure group (38.0000 ± 4.0497* versus* 32.6667 ± 4.1793 times, *P* > 0.05). However, there were no obvious changes in CF between ethyl acetate layer fraction of aqueous extract 40 g/kg and microwave exposure group.

### 3.2. Relative Reproductive Organ Weight

**Figure 3:**
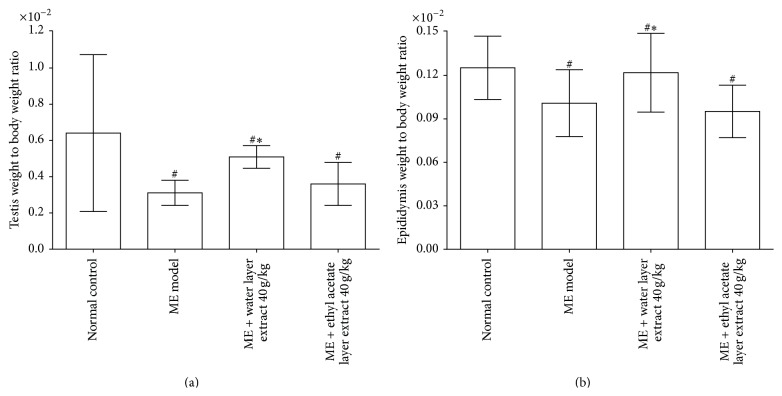
Effects of different parts of aqueous extract on testis to body weight ratio (a) and epididymis to body weight ratio (b). Compared with the microwave exposure model group, water layer fraction of aqueous extract 40 g/kg treated rats showed an increased testis to body weight ratio and epididymis to body weight ratio (all *P* < 0.05), but not compared with the ethyl acetate layer fraction of aqueous extract 40 g/kg treated rats. Values are expressed as mean ± SD (*n* = 6). ^#^ *P* < 0.05,* versus* normal control group. ^*∗*^ *P* < 0.05,* versus* microwave exposure model group. ME = microwave exposure.

As shown in Figures 3(a) and 3(b), significant decreases in the testis weight to body weight ratio and epididymis weight to body weight ratio were observed in the microwave exposure model compared with the normal control (all *P* < 0.05). Treatment with water layer fraction of aqueous extract 40 g/kg significantly increased testis-body weight ratio and epididymis-body weight ratio compared with those of the microwave exposure group (*P* < 0.05). However, no significant changes were observed in the reproductive organ to body weight ratio in the ethyl acetate layer fraction of aqueous extract 40 g/kg group.

### 3.3. Changes of Serum Levels of GnRH, FSH, LH, and Testosterone

**Figure 4:**
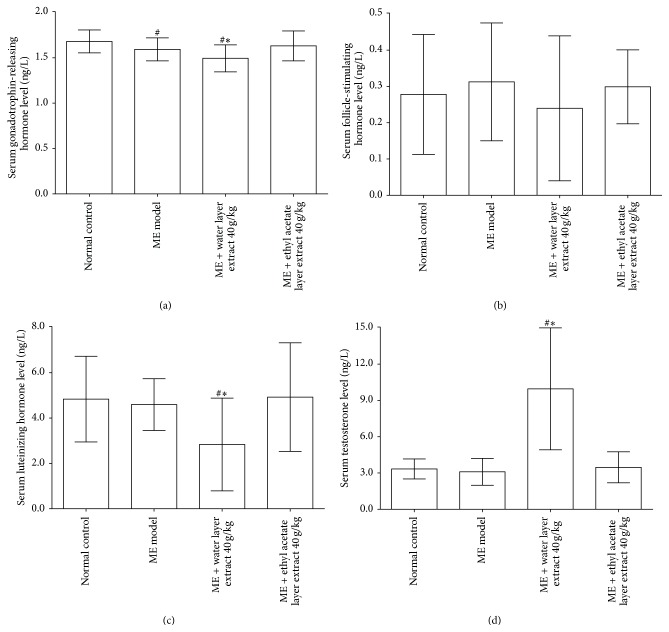
Effects of different parts of aqueous extract on serum levels of gonadotrophin-releasing hormone (a), follicle-stimulating hormone (b), luteinizing hormone (c), and testosterone (d). Values are expressed as mean ± SD (*n* = 6). ^#^ *P* < 0.05,* versus* normal control group. ^*∗*^ *P* < 0.05,* versus* microwave exposure model group. ME = microwave exposure.

As shown in Figure 4(a), significant decreases in serum GnRH levels were observed in the microwave exposure model compared with the normal control (1.5903 ± 0.0626* versus* 1.6838 ± 0.0603 ng/L; *P* < 0.05). Treatment with water layer fraction of aqueous extract 40 g/kg significantly decreased serum GnRH levels compared with the microwave exposure model group (1.4987 ± 0.0739* versus* 1.5903 ± 0.0626 ng/L; *P* < 0.05). As shown in Figure 4(b), treatment with water layer fraction of aqueous extract 40 g/kg did not significantly affect serum FSH levels compared with the microwave exposure model group (*P* > 0.05). As shown in Figures 4(c) and 4(d), compared with the microwave exposure model group, water layer fraction of aqueous extract 40 g/kg treatment was associated with a decrease in serum LH levels (2.8118 ± 1.0280* versus* 4.5915 ± 0.5609 ng/L; *P* < 0.05) and an increase in serum testosterone levels (9.8690 ± 2.5188* versus* 3.1262 ± 0.55885 ng/L, *P* < 0.05). However, ethyl acetate layer fraction of aqueous extract 40 g/kg did not affect serum levels of GnRH, FSH, LH, and testosterone.

### 3.4. Histopathological Changes of Testis and Epididymis

**Figure 5:**
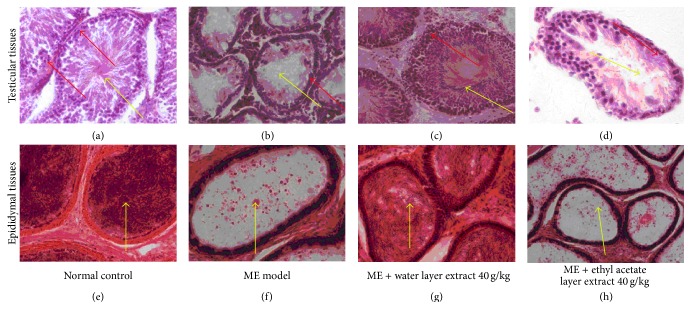
Effects of different parts of aqueous extract on histopathological changes in testis and epididymis. In testicular H&E staining (Figures 5(a)–5(d)), the normal control group showed well-arranged spermatogenic cell series (red arrow) and an abundance of sperms in the seminiferous tubule (yellow arrow) at 200x magnification; the microwave exposure model group showed wide spaces between each seminiferous tubule, a significant decrease in the seminiferous tubule diameter, fewer sperms in seminiferous tubule (yellow arrow), degeneration of spermatocyte/spermatogonia, and cell debris in ducts (red arrow) at 200x magnification; the water layer fraction of aqueous extract 40 g/kg group showed complete spermatogenic cell series and almost normal sperm counts at 200x magnification; the ethyl acetate layer fraction of aqueous extract 40 g/kg group showed necrosis and desquamate spermatogenic cells at 200x magnification. In epididymal H&E staining (Figures 5(e)–5(h) at 200x magnification), the normal control group showed an abundance of normal sperms in the epididymal ducts (yellow arrow); the microwave exposure model group showed no sperms except for many fallen spermatogenic cells or cell debris in the ducts (yellow arrow); the water layer fraction of aqueous extract 40 g/kg group showed increased quantity of sperms and rare exfoliated cells in the epididymal ducts (yellow arrow); the ethyl acetate layer fraction of aqueous extract 40 g/kg group showed only many exfoliated cells and no sperms in the epididymal ducts (yellow arrow).

Light microscopy of testicular sections taken from normal control testis showed normal seminiferous tubules containing developing sperm cells at different stages and interstitial tissue (Figure 5(a)). Testicular sections from microwave exposure model rats revealed various degrees of disorganization, degeneration, and reduction in germinal cells (Figure 5(b)). Testicular tissues from rats treated with a water layer fraction of aqueous extract 40 g/kg showed a well-preserved testicular histology (Figure 5(c)). In contrast, testicular sections in the ethyl acetate layer fraction of aqueous extract 40 g/kg group revealed various degrees of degeneration or necrosis of spermatogonia/spermatocyte (Figure 5(d)).

The epididymal tissues in normal control rats showed a normal architecture, characterized by an abundance of normal sperms in the epididymal ducts (Figure 5(e)). However, epididymal tissues in the microwave exposure group showed some exfoliated cells, cell debris, without sperm in the epididymal duct (Figure 5(f)). These findings were attenuated in the water layer fraction of aqueous extract 40 g/kg group and presented a great quantity of sperms (Figure 5(g)) while epididymal tissues in the ethyl acetate layer fraction of aqueous extract 40 g/kg group rats did not show obvious improvement, characterized by only a few exfoliated cells without sperms in the epididymal ducts (Figure 5(h)).

### 3.5. Immunohistochemical Analysis of GnRH

**Figure 6:**
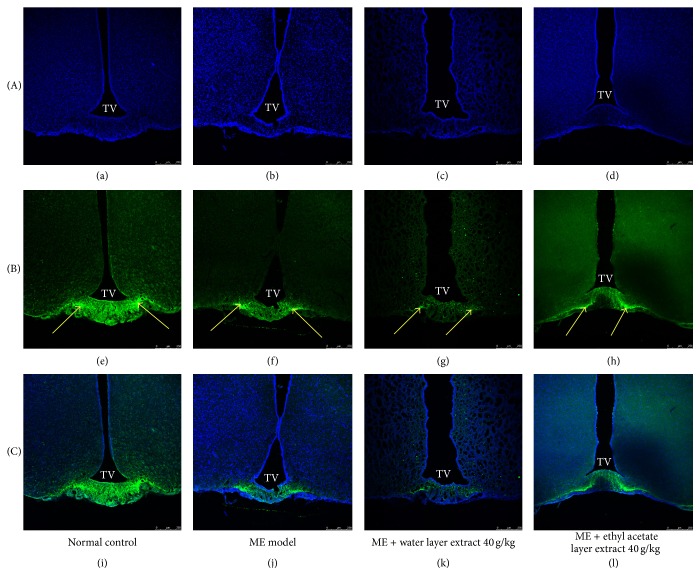
Representative coronal images of the arcuate nucleus of the hypothalamus showing immunohistochemical double labeling for GnRH/DAPI. Photomicrographs of DAPI ((a)–(d)) and GnRH ((e)–(h)) and merged images of DAPI and GnRH ((i)–(l)). The yellow arrow represents positive GnRH expression. Treatment with water layer fraction of aqueous extract 40 g/kg significantly decreased the GnRH fluorescence intensity of the hypothalamus compared with the microwave exposure group. GnRH = gonadotrophin-releasing hormone; TV = third ventricle. DAPI = 4,6-diamidino-2-phenylindole. Scale bar = 250 *μ*m.

Dual labeling immunofluorescence staining of hypothalamic sections revealed GnRH distribution in the arcuate nucleus of the hypothalamus below the third ventricle (Figures 6(a)–6(i)). The positive immunofluorescence density of GnRH was decreased in the microwave exposure group compared with the normal control group. The positive immunofluorescence density of GnRH was obviously decreased in the water layer fraction of aqueous extract treated rats but not in the ethyl acetate layer fraction of aqueous extract treated rats.

**Figure 7:**
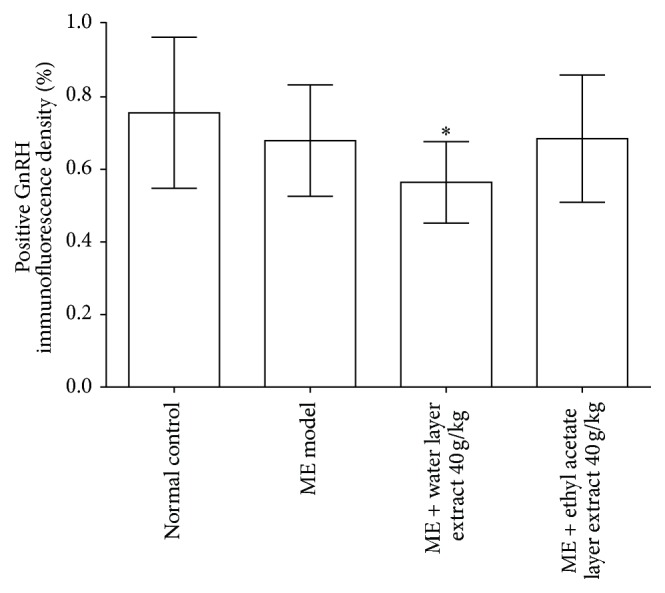
Percentage of positive GnRH immunofluorescence density. Values are expressed as mean ± SD (*n* = 6). ^#^ *P* < 0.05,* versus* normal control group. ^*∗*^ *P* < 0.05,* versus* microwave exposure model group. ME = microwave exposure. GnRH = gonadotrophin-releasing hormone.

As shown in Figure 7, despite the finding that microwave exposure showed some trend towards decreased GnRH protein expression, there was no statistically significant difference in the positive GnRH immunofluorescence density compared with the normal control group (0.6833 ± 0.0756* versus* 0.7605 ± 0.1035; *P* > 0.05). Treatment with water layer fraction of aqueous extract 40 g/kg significantly decreased positive GnRH immunofluorescence density compared with the microwave exposure group (0.5675 ± 0.0565* versus* 0.6833 ± 0.0756; *P* < 0.05). Ethyl acetate layer fraction of aqueous extract 40 g/kg did not affect positive GnRH immunofluorescence density in the hypothalamus.

## 4. Discussion

In this study, we established a reproductive impairment rat model by exposure to 900 MHz microwave with microwave signal generator at 218 *μ*m/cm^2^ radiation densities for consecutive 10 days. Male rat reproductive impairment induced by microwaves was confirmed by various degrees of degeneration or decreased number of spermatogonia/spermatocyte upon histological examination, reduction in the weight of testis and epididymis, and serum testosterone levels, as well as lower sexual performance.

Treatment with water layer fraction of aqueous extract derived from* Morinda officinalis F. C. How* 40 g/kg improved sexual performance, characterized by decreased CIP; increased the weight of reproductive organs; attenuated histopathological changes of testis and epididymis; increased serum testosterone; decreased serum LH and GnRH levels; and decreased GnRH positive immunofluorescence density. Together these findings suggest that water layer fraction of aqueous extract from* Morinda officinalis F. C. How* in the doses used in this study can ameliorate microwave-reduced hypothalamic-pituitary-testicular impairment.

Male reproductive function is a complex process requiring high energy. Testis is the central organ of the male germline regulated by the hypothalamus and pituitary through different feedback regulation mechanisms. Regulatory mechanisms are controlled mainly through GnRH, FSH, and LH acting on the hypothalamus-pituitary-testis axis. The hypothalamus monitors reproductive state through neurons that release GnRH. GnRH promotes LH and FSH release through the anterior pituitary. LH acts on testicular interstitial cells, promoting secretion of testosterone. FSH is responsible for the activation of the seminiferous tubules maintaining the production of sperm. Therefore, hypothalamic injury indirectly leads to male reproductive impairment and further results in injury of reproductive organs. Meanwhile, reproductive organ damage can induce disorder of hypothalamic-pituitary-testicular axis through negative feedback mechanisms [19].

The possible adverse effects of exposure to cell phone microwaves on male reproductive disorder have been a hot topic. Evidence from human and animal studies suggests a possible link between cell phone use and male infertility [20]. Mobile phones are often carried in trouser pockets or belt clip near the reproductive organs in men. Carrying a mobile phone near the testes may result in decreased testosterone or sperm production in men. Findings from our study were in line with many studies. Ozguner et al. [21] indicated that the diameter of seminiferous tubules and the mean height of the germinal epithelium were significantly decreased after 900 MHz microwave exposure in male rats. Ghanbari et al. [22] found that exposure to cell phone waves 950 MHz for 14 days decreased sperm viability and motility in rats. Meo et al. [23] reported that exposure to mobile phone radiation for 60 min/day for up to 3 months significantly reduced serum testosterone levels. More recently, Odacı and Özyılmaz [24] showed that exposure to a 900 MHz electromagnetic field for 1 h/day over 30 days caused bad outcomes in adult rat testicular morphology and biochemistry. In human studies, an obvious decrease in sperm motility in semen samples exposed to a 900 MHz cell phone for 5 min was observed among men [25]. Mobile phone radiation exposure was associated with DNA fragmentation and decreased sperm motility in healthy men [26, 27]. In addition, cell phone use in men was associated with decreased sperm count and viability [28, 29], which may contribute to male infertility. In contrast to above studies and our study, few studies [30, 31] have reported no adverse effects of 900 MHz microwave exposure on testicular function or structure and epididymal sperm counts in male rats.

Testosterone is primarily a male hormone and reduction in testosterone level may result in reproductive disorder. Male reproductive function is regulated by precise and coordinated secretion of GnRH, LH, FSH, and testosterone. Testes are responsible for the production of spermatozoa and synthesis and release of testosterone. In the current study, after water layer fraction of aqueous extract 40 g/kg treatment for 14 days, serum levels of testosterone significantly increased and LH decreased significantly compared with the values found in microwave exposure model rats. However, serum levels of LH and FSH were differentially modified, which indicated that water layer fraction of aqueous extract differentially affects both hypothalamic and pituitary levels in the hypothalamic-pituitary-testicular axis. The increase in serum testosterone levels observed after water layer fraction of aqueous extract treatment may reflect direct effects on the testis. Testosterone acts on the seminiferous tubules to initiate and maintain spermatogenesis [32]. Our study was supported by Liu et al. who indicated that treatment with water decoction of* Morinda officinalis F. C. How* 20 g/kg significantly increased serum testosterone levels compared with microwave exposure model rats [33]. Histopathological analysis demonstrated a significant increase in the epididymal sperm count of rats treated with a water layer fraction of aqueous extract for 14 days along with higher serum testosterone levels. In addition, increment in the weight of testis and epididymis may be attributed to increased secretory activity of these organs.

The positive GnRH immunofluorescence density of the arcuate nucleus in the hypothalamus was significantly decreased in the water layer fraction of aqueous extract treated rats. Meanwhile, serum levels of GnRH were also reduced. These results might be explained by the water layer fraction of aqueous extract directly increasing testosterone level and indirectly inhibiting GnRH synthesis and release* via* a feedback loop system. Taken together, the protective effect of* Morinda officinalis F. C. How* on reproductive impairment is mainly located at the testicular and epididymal level while changes of GnRH, LH, or FSH might be regulated by gonadal hormone feedback loop system involving the anterior pituitary and hypothalamus and testis.

*Morinda officinalis How*, as a Kidney-Yang tonic agent, has been used to enhance sexual functions for thousands of years.* Morinda officinalis F. C. How* decoction could decrease sperm deformity rate in male mice* via* acting on spermatogonia and primary spermatocytes [34]. Bajijiasu, isolated from the roots of* Morinda officinalis F. C. How*, either enhanced the sexual function of normal mice or increased testosterone levels and histopathological findings in the testis [35].* Morinda officinalis F. C. How* oligosaccharides promoted the growth of the testis and increased production of sperm by the testis [36]. These findings suggest that crude* Morinda officinalis F. C. How* and its extract had therapeutic potential for male infertility.

Oxidative stress, a cellular or physiological condition, plays an important role in male infertility. A possible link between the biological systems and microwave radiation is oxidative stress. Microwave radiation is responsible for the generation of free radicals and reactive oxygen species, which might be a possible link between male reproductive impairments and microwave exposure. Nonthermal effects of cell phones lead to oxidative stress on the male reproductive system through increased generation of seminal reactive oxygen species and reduction in antioxidant enzymes [37]. The extract of* Morinda officinalis* has shown antioxidant properties [38, 39]. Acidic polysaccharide from* Morinda officinalis F. C. How* may partly contribute to its antioxidant activity [40].* Morinda officinalis F. C. How* protected functions of cultured mouse TM3 Leydig cells from hydrogen peroxide-induced oxidative stress* in vitro* [41]. Taken together, antioxidant properties of* Morinda officinalis F. C. How* may be one of the possible mechanisms against microwave-induced reproductive impairments.

There were many limitations in the current study. First, antioxidant treatment could be beneficial in preventing or decreasing some complications of microwave radiation [42, 43]; however, whether the protective effects of water layer fraction of aqueous extract of* Morinda officinalis F. C. How* occur* via* antioxidant property needs to be further studied. Second, we did not investigate the effects of water layer fraction of aqueous extraction impairment on sperm. Additional future studies should be focused on the assessment of the protective effects on sperm injury both* in vitro* and* in vivo*. Finally, this study cannot determine the dose-effect of aqueous extract of* Morinda officinalis F. C. How*. A future study is suggested to investigate the dose-effect of aqueous extract of* Morinda officinalis F. C. How*.

In conclusion, the present study demonstrated that a water layer fraction of aqueous extract derived from* Morinda officinalis F. C. How* had protective effects against hypothalamus-pituitary-testis axis impairment induced by microwave radiation in male rats. This study thus provided some evidence that* Morinda officinalis F. C. How* might be a promising agent for treatment of male reproductive impairment induced by microwave radiation. However, detailed molecular mechanisms of action and key active compounds of* Morinda officinalis F. C. How* still need to be further investigated.
